# Multi-Interactions in Ionic Liquids for Natural Product Extraction

**DOI:** 10.3390/molecules26010098

**Published:** 2020-12-28

**Authors:** Ying Zhang, Yingying Cao, Hui Wang

**Affiliations:** 1 Beijing Key Laboratory of Ionic Liquids Clean Process, Institute of Process Engineering, Chinese Academy of Sciences, Beijing 100190, China; zhangying17@ipe.ac.cn (Y.Z.); yycao@ipe.ac.cn (Y.C.); 2 CAS Key Laboratory of Green Process Engineering, Institute of Process Engineering, Chinese Academy of Sciences, Beijing 100190, China; 3 State Key Laboratory of Multiphase Complex Systems, Institute of Process Engineering, Chinese Academy of Sciences, Beijing 100190, China; 4 Innovation Academy for Green Manufacture, Chinese Academy of Sciences, Beijing 100190, China; 5 School of Chemical Engineering, University of Chinese Academy of Sciences, Beijing 100049, China

**Keywords:** ionic liquids, multi-interactions, extraction, natural products

## Abstract

Natural products with a variety of pharmacological effects are important sources for commercial drugs, and it is very crucial to develop effective techniques to selectively extract and isolate bioactive natural components from the plants against the background of sustainable development. Ionic liquids (ILs) are a kind of designable material with unique physicochemical properties, including good thermal stability, negligible vapor pressure, good solvation ability, etc. ILs have already been used in pharmaceuticals for extraction, purification, drug delivery, etc. It has been reported that multi-interactions, like hydrogen bonding, hydrophobic interactions, play important roles in the extraction of bioactive components from the plants. In this review, recent progress in the understanding of scientific essence of hydrogen bonding, the special interaction, in ILs was summarized. The extraction of various natural products, one important area in pharmaceutical, by conventional and functional ILs as well as the specific roles of multi-interactions in this process were also reviewed. Moreover, problems existing in bioactive compound extraction by ILs and the future developing trends of this area are given, which might be helpful for scientists, especially beginners, in this field.

## 1. Introduction

With the improvement of living standards and the strengthening of health awareness, people are paying more attention to natural products, which could be extracted from plants and exhibit special physiological effect [1,2,3,4]. Natural products have been widely applied in food, healthcare, perfume, cosmetics and pharmaceutical industries [5,6,7,8]. For instance, natural products are a significant source of new drugs. To date, a huge number of commercial drugs used in the clinic are derived from natural products [9]. Therefore, the extraction and separation of natural products from plants are becoming more and more significant, and have attracted more attention from academia and industry.

The conventional ways for natural products separation include ultrasonic-assisted extraction, microwave-assisted extraction, heat reflux extraction, and soxhlet extraction, etc. [10,11,12]. The solvents involving in this process are volatile/explosive organic compounds like ethanol, methanol, petroleum ether, ethyl acetate, benzene. For example, ultrasonic-assisted extraction by ethanol solution was used to extract wedelolactone and antioxidant polyphenols from *Eclipta prostrate* L. Under the optimum conditions, the extraction amounts of wedelolactone and total phenolic compounds were 3.90 mg/g and 22.57 mg/g, respectively [13]. The traditional volatile and hazardous organic solvents employed in the extraction of natural products have drawbacks, including a large consumption of solvents, environmental pollution, low extraction selectivity towards the target compounds, high energy input in solvent recovery, etc. Consequently, it would be highly desirable and strategic to develop environmentally benign solvents for natural product production.

ILs are a kind of organic salts in liquid state below 100 °C which consists of an organic cation paired with an inorganic or organic anion [14]. As alternative media, ILs are well recognized due to their excellent properties including high thermal stability, low vapor pressure, low volatility, good solvation ability, etc. [15,16]. Importantly, compared with traditional molecular solvents, the physicochemical properties of ILs can be finely tuned through modification of the structures of the cation and/or anion, thus making it possible to design task specific ILs according to the requirements of certain processes [17,18]. ILs have been widely applied in extraction and separation, organic synthesis, chromatographic analysis, electrochemistry, catalysis, natural polymer dissolution and processing (e.g., starch), etc. [19,20,21,22].

It has been demonstrated that ILs are promising extraction solvents for natural products [23,24]. Strehmel et al. [25] studied the extraction of active components from the bark of *Betula pendula* by 1-butyl-3-methylimidazolium acetate, and the methanol insoluble part of trunk could also be separated by this IL, which is superior to extraction by traditional solvents like methanol. Jiang et al. [26] extracted wheat-esterase from wheat using aqueous biphasic systems (ABS) formed by NaH_2_PO_4_ and 1-butyl-3-methylimidazolium tetrafluoroborate ([C_4_mim][BF_4_]) solution. The result showed that ABS composed of 20% [C_4_mim][BF_4_] (*w*/*w*) and 25% (*w*/*w*) NaH_2_PO_4_ (pH = 4.8) exhibited good selectivity for wheat-esterase, and the yield of wheat-esterase could reach 88.9%. Both the purity and yield of wheat-esterase were enhanced compared with those obtained by the conventional NaH_2_PO_4_ salting-out process. The extraction process using ILs as the solvents exhibits advantages including higher extraction efficiency, mild conditions, negligible VOC emissions and easy recyclability. Moreover, ionic liquids can interact with the target compounds, resulting in high selectivity.

The excellent performance of ILs in chemical processes, including natural product extraction, is closely related to the microstructure in IL media, which is determined by the ions and their specific interactions [27]. Obviously, ILs are composed of anions and cations so electrostatic forces are important in ILs [16]. However, reports have demonstrated strong evidence for the existence of hydrogen bonding and their importance in the performance of ILs [28]. Hydrogen bonding is a kind of strong directional interaction compared to other forces in liquid molecules, e.g., hydrophobic or solvophobic interactions, and is therefore regarded as a kind of special interaction in ILs.

Although there are many studies on the extraction of natural products by ionic liquids [29], to the best of our knowledge, comprehensive review on the role of interactions in ILs, such as the cation-anion interactions, hydrogen bonding, hydrophobic interactions, on natural product extraction has not been reported. In this review, recent progress on the special interaction in ILs, i.e., hydrogen bonding, and the derivative ionic pairing, stacking, and self-assembling in ILs were summarized. The extraction of various natural products using ILs and the roles of multi-interactions in the extraction process were also reviewed. Problems in this extraction process were analyzed and future developing trends were given.

## 2. Hydrogen Bonding in Ionic Liquids

The special physicochemical properties of ILs depend on the specific interactions between the cations and anions, as well as the formative microstructure in the system. These interactions range from weak solvophobic, van der Waals, dispersion forces to strong hydrogen bonding, electron pair donor/acceptor interactions, Coulombic forces, magnetic dipole, halogen bonding, which lead to the unique properties/behaviors of this kind of fantastic materials. Hydrogen bonding has been reported to be one of the most important interactions in ILs, which has drawn research attention of scientists in recent years. In this section, the research progress in understanding of hydrogen bonding in ILs is reviewed.

**Figure 1:**
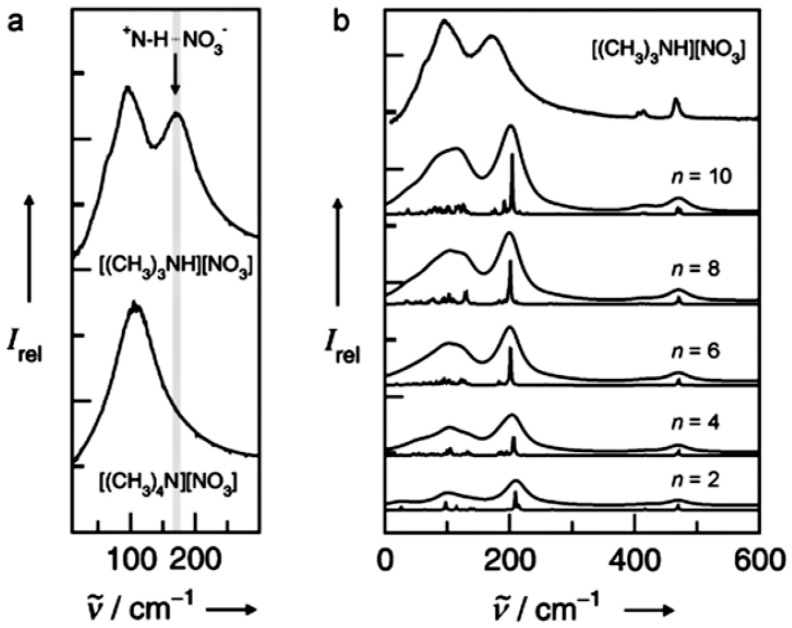
(**a**) Collected FIR spectra of the protic ionic liquids [(CH_3_)_3_NH][NO_3_] (top) and [(CH_3_)_4_N][NO_3_] (bottom). (**b**) Calculated FIR spectra of clusters of ion pairs of {[(CH_3_)_3_NH][NO_3_]}_n_ with *n* = 2, 4, 6, 8, 10 [32]. Reprinted with permission of Wiley.

Ion pairs (i.e., cation-cation, cation-anion or anion-anion) as the repeating units in ILs determine their properties. When the ILs cations contain hydrogen atoms and the anions have lone pairs, there is a potential to form hydrogen bonding [30]. Dong et al. [31] first demonstrated the existence of hydrogen bonding of ion pairs in imidazolium ionic liquids. Spectroscopic techniques (e.g., IR, NMR), density functional theory (DFT) and ab initio molecular dynamics (AIMD) simulations have been used for the exploration of hydrogen bonding in ILs. Experimentally, hydrogen bonding interactions can be tested by far-infrared spectroscopy, which was reported in detail by Fumino et al. [32] The far-infrared spectra of trimethylammonium nitrate ([(CH_3_)_3_NH][NO_3_]) and tetramethylammonium nitrate ([(CH_3_)_4_N][NO_3_]) were collected. For [(CH_3_)_4_N][NO_3_], a broad absorption peak around 100 cm^−1^ was observed, which was attributed to the vibrational contributions of interacting ions. A similar peak around 100 cm^−1^ and a distinct vibrational peak at 171 cm^−1^ were observed in the spectrum of ([(CH_3_)_3_NH][NO_3_]) (Figure 1a). The additional peak at 171 cm^−1^ was ascribed to the ^+^N-H···NO_3_^−^ hydrogen bonding. Based on the results, it can be concluded that quaternary ammonium salts with protic hydrogen atoms are easy to form hydrogen bonding. The spectra were also simulated and the calculated FIR spectra of ion pairs clusters in {[(CH_3_)_3_NH][NO_3_]}_n_ are shown in Figure 1b. The simulated spectra matched well with the measured one (i.e., the top one). In the calculation, the main vibrational bands are better separated than those in the measured one, which could be due to the matrix effect in the IL. Theoretical studies based on simulation, including ab initio calculations, Car-Parrinello molecular dynamics simulations as well as classical molecular dynamics simulations, have also been employed to reveal the interactions between ions in ILs. Combining these simulations with various spectroscopic characterizations could further prove the existence of hydrogen bonding interactions in ILs [33,34]. Zentel et al. [35] utilized density functional-based tight-binding (DFTB) and DFT to study the infrared spectroscopic and dynamics signals of hydrogen bonding in ethylammonium nitrate salt. A broader distribution of the CH- and NH- stretching bands were indicated by DFTB, demonstrating the existence of the N-H···O hydrogen bonding. Thus, the -NH groups and oxygen atoms can form an asymmetric network.

**Figure 2:**
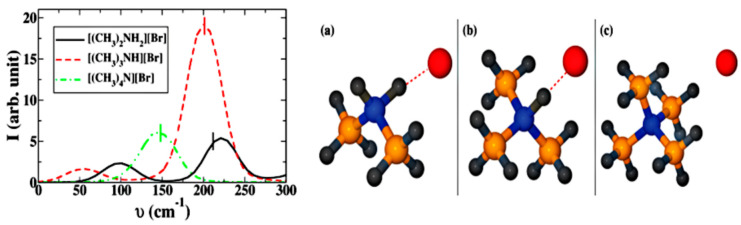
Far-IR spectra (left) and ion pairs geometries (right) of three alkylammonium salts: (**a**) [(CH_3_)_2_NH_2_]Br, (**b**) [(CH_3_)_3_NH]Br, and (**c**) [(CH_3_)_4_N]Br [40]. Reprinted with permission of American Chemical Society.

**Figure 3:**
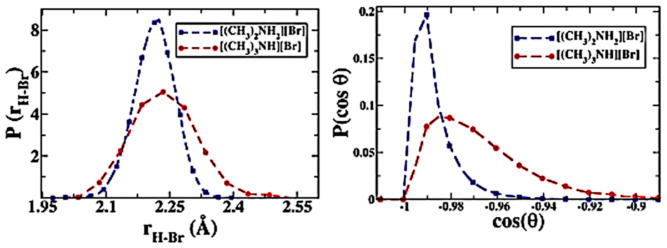
Normalized distribution of NH···Br bonding length (left) and N−H···Br angle (right) in [(CH_3_)_3_NH]Br and [(CH_3_)_2_NH_2_]Br [40]. Reprinted with permission of American Chemical Society.

There also exists hydrogen bonding between anion and cation in task specific ILs such as halogenated quaternary ammonium ionic liquids (i.e., *N*-trimethyl-*N*-propylammonium bis(trifluoromethylsulfonyl)imide ([N_3111_][NTf_2_]), bihydroxyethylbiethylammonium bromide ([NEt_2_(HE)_2_]Br)), amino acid-based ionic liquids (i.e., L-proline trifluoroacetate ([L-Pro][CF_3_COO]), L-proline sulfate ([L-Pro]_2_[SO_4_])), protic ionic liquids (i.e., [(CH_3_)_3_NH][NO_3_], dimethylammonium bromide ([(CH_3_)_2_NH_2_]Br)), etc. In these ILs, the cation and anion contain proton acceptor and donor sites, resulting in formation of an extended hydrogen bonding network [36,37,38]. The hydrogen bonding between the anion and cation expressed as ^+^[A–H···B]^−^ shows particular features in the geometric, electronic, and dynamic aspects, which is inherently different from that of the conventional hydrogen bonding in molecular solvents expressed as A–H···B [39]. Balasubramanian et al. [40] used DFT and AIMD simulations to study the vibrational signals of cation-anion hydrogen bonding in protic ILs. In FTIR spectra, the vibration signals of hydrogen bonding were detected in the range of 150–240 cm^−1^, as shown in Figure 2 (left). With the increase of the number of hydrogen bonding on the cation, blue shift in the FTIR was observed and the exact peak position can be adjusted by the strength of the cation-anion hydrogen bonding. The AIMD trajectories were utilized to calculate the bond length and angle in [(CH_3_)_4_N]Br, trimethylammonium bromide ([(CH_3_)_3_NH]Br) and [(CH_3_)_2_NH_2_]Br, and results are presented in Figure 2 (right). The cation-anion hydrogen bonding in [(CH_3_)_2_NH_2_]Br with the N−H···Br angle of 180° is more linear than that (165°) in [(CH_3_)_3_NH]Br (as shown in Figure 3), suggesting stronger intermolecular interactions in the former IL. The calculated NMR results were also in accordance with the vibrational analysis. Ossowicz et al. [41] found that when the imidazolium cation had a chiral center or the anion is halide, the hydrogen bonding could expand in space to form a 3D hydrogen bonding network, which could stabilize the conformation of a single ion to affect the properties of ILs [42].

The structures of anions in ILs closely relate to the physicochemical properties and different types of anions present different hydrogen bonding donating/accepting ability. There only exists one-hydrogen-bonding ion pair in ILs with single atomic anion (e.g., Br^−^ or Cl^−^), and multiple atomic anions (e.g., [BF_4_]^−^ or [PF_6_]^−^) can form one- or more-hydrogen-bonding ion pairs [43]. Li et al. [44] designed and prepared six hydroxyl-functionalized imidazolium ILs with different anions ([DCA]^−^, [PF_6_]^−^, [NO_3_]^−^, [NTf_2_]^−^, [BF_4_]^−^ and [SCN]^−^). Their absorption capacities for NH_3_ ranked in descending order of: [NTf_2_]^−^ > [PF_6_]^−^ > [BF_4_]^−^ > [SCN]^−^ > [NO_3_]^−^. The fluoride in the anions and hydrogen in NH_3_ can form hydrogen bonding, so that ILs containing fluoride have higher absorption capacity. One [BF_4_]^−^ anion has four acceptors, [PF_6_]^−^ has six acceptors, and [NTf_2_]^−^ has ten acceptors, thus, hydrogen in NH_3_ and [NTf_2_]^−^ can form stronger hydrogen bonding, leading to the highest solubility of NH_3_ in 1-(2-hydroxyethyl)-3-methylimidazolium bis(trifluoromethylsulfonyl)imide ([EtOHmim][NTf_2_]). Shang et al. [45] studied the NH_3_ dissolving capability of three types of ILs with cations of various hydrogen bonding donating abilities, i.e., 1-*n*-butyrate-3-methylimidazolium bis(trifluoromethylsulfonyl)imide ([HOOC(CH_2_)_3_mim][NTf_2_]), 1-butylimidazolium bis(trifluoromethylsulfonyl)imide ([Bim][NTf_2_]) and 1-butyl-3-methylimidazolium bis(trifluoromethylsulfonyl)imide ([C_4_mim][NTf_2_]). The solubility of NH_3_ in these three ILs at 40 °C increased in the order of: [C_4_mim][NTf_2_] < [HOOC(CH_2_)_3_mim][NTf_2_] < [Bim][NTf_2_]. The fact that [Bim][NTf_2_] possessed the highest NH_3_ absorption capacity was attributed to the existence of hydrogen bonding interactions between the NH_3_ molecule and the protic proton on the imidazole ring.

**Figure 4:**
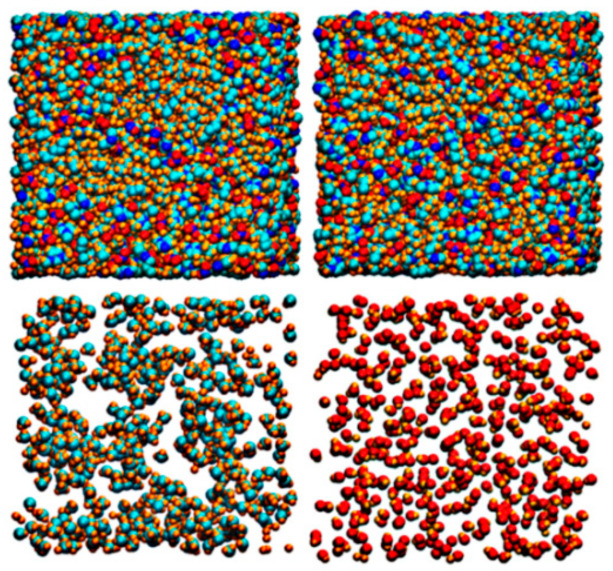
Random snapshots of C_8_ (left column) and C_OH_ (right column). First row: all atoms; second row: terminal groups (CH_3_ for C_8_ and OH for C_OH_) only. Carbon atoms are colored with cyan, nitrogen with blue, oxygen with red, and hydrogen with gold [47]. Reprinted with permission of Elsevier.

In principle, cation-cation attractions could also exist in ILs where the cation is substantial charge delocalized and the anion is weakly coordinating. For instance, the quaternary ammonium and imidazolium cations can form cation-cation hydrogen bonding. Ludwig et al. [46] studied the interactions of ions in 1-(2-hydroxyethyl)-3-methylimidazolium tetrafluoroborate ([EtOHmim][BF_4_]). It was found that new infrared bands at 3547 cm^−1^ in the -OH frequency range appeared at low temperatures, indicating the formation of OH^…^O hydrogen bonding in cation-cation clusters, which was also supported by DFT calculations. Deng et al. synthesized 1-octyl-3-methylimidazolium nitrate (denoted as C_8_) and 1-(8-hydroxyoctyl)-3-methylimidazolium nitrate (denoted as C_OH_) [47], and discovered that the hydroxyl groups in the cation could form hydrogen bonding with other groups (e.g., oxygen in the [NO_3_]^−^ anion, the hydroxyl groups in the imidazolium cation), as shown in Figure 4. The amphiphilic ionic liquid C_8_ could be transformed to a hydrophilic one by the introduction of hydroxyl group. In addition, owing to the great number of hydrogen bonding, the molecular kinetics of the IL system with a hydroxyl tail is slower than the one with an alkyl terminal, as indicated by the self-diffusion of all ions and lifetime of ion cages.

The hydrogen bonding network in ILs can enhance the viscosity, which reduces mass transfer and would influence their performance in a reaction/separation process. The miscible co-solvent added into ILs can weaken the interactions between anion and cation, which could destroy the hydrogen bonding network and the aggregation of IL ions, thus greatly decreasing the viscosity. In IL-organic solvent mixtures, the ion-pairs are assembled together to form aggregates or clusters, which are of different sizes, including angstrom scale, nanoscale, the bulk, etc. [48,49,50,51]. Yu et al. [52] added acetonitrile (CH_3_CN) into [C_4_mim][BF_4_], and the interactions were analyzed in detail by ATR-IR, ^1^H NMR and DFT. The results showed that CH_3_CN molecules can destroy the ion clusters into pairs within the concentration range investigated (the mole fractions of CH_3_CN in [C_4_mim][BF_4_]-CH_3_CN mixtures were 0.11, 0.19, 0.32, 0.41, 0.50, 0.60, 0.70, 0.80, and 0.90), and the strength of hydrogen bonding between [C_4_mim]^+^ and the N of acetonitrile was enhanced with the CH_3_CN concentration increasing.

The hydrogen bonding between the ions in ILs is closely accompanied by the electrostatic attractions as ILs are composed of ions, and both of them together form a new type of interactions or nanoclusters to decide the structure directionality. To more accurately express the interactions in ILs, the coupled interactions of hydrogen bonding and electrostatic attractions in ILs are defined as *Z*-bonds by Dong and co-workers [53]. *Z*-bonds can be commonly utilized to describe the interactions between the cation and anion in ILs, and many bio-systems relating to ions, ionic reaction, and ionic materials.

The hydrogen bonding interactions which can be tuned by the structure of the cation and/or anion combinations play critical roles in ILs application, including extraction and separation. Fan et al. [54] utilized imidazolium-based ILs to separate hydroxycinnamic acid from water. It was found that the imidazolium-based ILs with the trifluoromethanesulfonate ([CF_3_SO_3_]^−^) anion showed high extraction capacity for the acid due to its strong hydrogen bonding ability with hydroxyl group in hydroxycinnamic acid. Furthermore, the incorporation of hydroxyl on the cation could improve the extraction efficiency, and the IL [C_4_C_11_OHim][CF_3_SO_3_] had the strongest extraction capability for hydroxycinnamic acid.

## 3. Extraction of Natural Products Using Ionic Liquids

### 3.1. Conventional Ionic Liquids

Conventional ILs are the ones without functional groups, which are usually the first choice when this kind of fantastic materials is proposed to be used in a certain field, including natural product extraction. The structures of ILs have an important impact on the physicochemical properties, which are closely related to the extraction efficiency for target compounds. It has been shown that both the cation and anion can affect the extraction efficiency. In this subsection, influence of the structure of the cations and anions on the extraction efficiency will be discussed. It should be mentioned that the analytical techniques used in IL-extraction for quantification of natural products include thin layer chromatography, high performance liquid chromatography, countercurrent chromatography, and so on.

#### 3.1.1. Effect of the Cation

On the basis of the type of cations, ILs can be mainly divided into the following kinds, e.g., imidazolium, piperrolium, pyrrolinium, pyridinium, ammonium, and phosphonium ionic liquids, etc. [55]. Peng et al. synthesized several ILs, by pairing the 1-dodecyl-3-methylimidazolium ([C_12_mim]^+^), methylethylmorpholinium ([Memo]^+^), [C_2_mim]^+^, butylmethylpyrrolidinium ([Bmpyr]^+^), ethylpyridinium ([EPy]^+^) or methylethylpiperidine ([Mepip]^+^) cation with the same Br^−^ anion, for the separation of organic and inorganic iodine from *Laminaria*. The results showed that [EPy]Br was the optimum extracting agent. This may be due to the stronger multiple interactions of [EPy]Br solution with iodine analytes (monoiodo-tyrosine and diiodo-tyrosine), including hydrogen bonding, ion/charge-charge and π-π interactions [56]. Sun et al. employed ionic liquid-based enzyme-assisted method to extract chlorogenic acid (CGA) from *Flos Lonicera Japonica* [57]. The maximum extraction efficiency was 93.35% under the optimal conditions of 45.86% (*w*/*w*) IL and 8.27% (*w*/*w*) Na_2_SO_4_ at 22 °C and pH of 11.0, which may be due to the hydrogen bonding and hydrophobic interactions between ionic liquids and CGA. The coupled method of ionic liquid-lithium salt based microwave pretreatment and ultrasonic-assisted extraction was proposed to extract syringin and oleuropein from *Syringa reticulata var. mandshurica* branch bark [58]. The IL 1-butyl-3-methylimidazolium bromide exhibited the best performance for the extraction. Under the optimal extraction conditions in the system of [C_4_mim]Br-lithium salt, the extraction amounts of syringin and oleuropein were 0.75 ± 0.05 mg/g and 5.46 ± 0.22 mg/g, respectively. Disruption of the intermolecular hydrogen bonds of the plant cellulose by the ionic solvent would dissolve the cell wall and release the target compounds. Therefore, higher syringin and oleuropein yields were observed in [C_4_mim]Br–lithium salt system.

**Figure 5:**
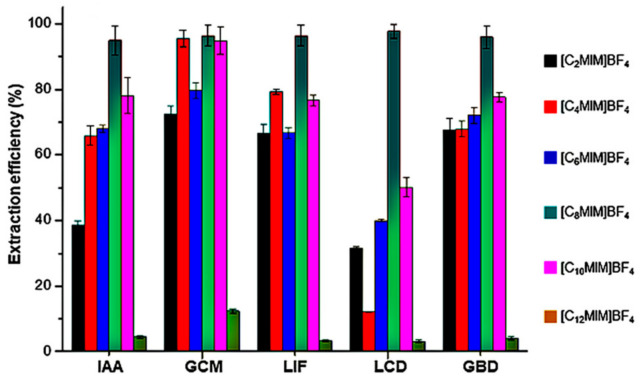
Influences of different ILs on extraction efficiency of GBD, LCD, LIF, GCM and IAA from licorice [59]. Reprinted with permission of Elsevier.

In addition, the changes of alkyl chain length on the cations have an important effect on extraction. Ji et al. [59] investigated the extraction of prenylated flavonoids: glabridin (GBD), licoricidin (LCD), licoisoflavanone (LIF), glycycoumarin (GCM) and isoangustone A (IAA) using six 1-alkyl-3-methylimidazolium ionic liquids, namely [C_12_mim][BF_4_], [C_10_mim][BF_4_], [C_8_mim][BF_4_], [C_6_mim][BF_4_], [C_4_mim][BF_4_] and [C_2_mim][BF_4_]. The results in Figure 5 illustrated that the extraction efficiency of GBD, LCD, LIF, GCM and IAA increased with the increasing of side alkyl chain length from ethyl to octyl, which could be probably attributed to the enhanced hydrophobic interactions with increase of the alkyl chain length as the prenylated flavonoids are hydrophobic. Owing to the obvious increase in viscosity of ionic liquids, the extraction efficiency decreased with the cations further changing from octyl to dodecyl. Therefore, [C_8_mim]^+^ was proven to be the optimum cation to extract prenylated flavonoids.

#### 3.1.2. Effect of the Anion

**Figure 6:**
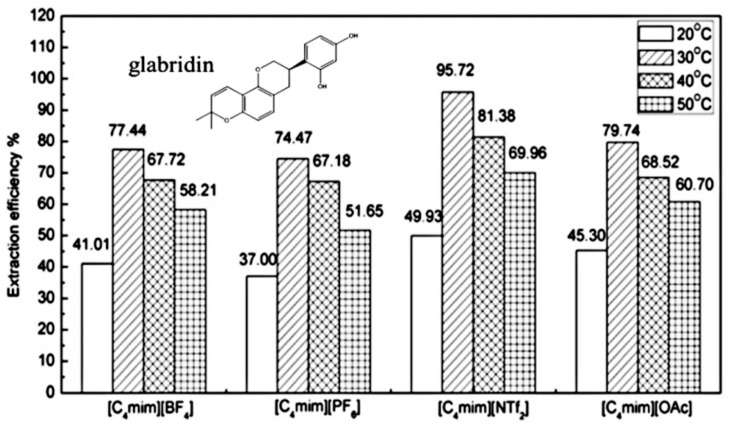
Effect of IL anions on extraction efficiency for glabridin [61]. Reprinted with permission of Elsevier.

According to the type of the anions, ILs can be classified into acidic, neutral and basic ones. Fan et al. synthesized 1-butyl-3-hexylimidazolium based hydrophobic ionic liquids with different anions ([ClO_4_]^−^, [BF_4_]^−^, [PF_6_]^−^, [CF_3_SO_3_]^−^ and [NTf_2_]^−^) to evaluate their extraction capacities for gramine and quinine [60]. It was found that the extraction efficiency mainly relied on the hydrogen bonding strength as well as hydrophobicity of the IL anion. The 1-butyl-3-hexylimidazolium IL with [CF_3_SO_3_]^−^ anion, showed better extraction capacities for the two alkaloids. It was considered that F and O in [CF_3_SO_3_]^−^ could form hydrogen bonding with the proton in -NH group of gramine or -OH group of quinine. Li et al. studied the extraction of bioactive glabridin from licorice using ILs with the same [C_4_mim]^+^ cation and different anions, including [OAc]^−^, [NTf_2_]^−^, [BF_4_]^−^ and [PF_6_]^−^ [61]. The results showed that the IL anions had an important influence on extraction capacities for glabridin, which increased in the order of: [PF_6_]^−^ < [BF_4_]^−^ < [OAc]^−^ < [NTf_2_]^−^, as shown in Figure 6. [C_4_mim][NTf_2_] showed the highest extraction capacity of 95.7% for glabridin. Each of the [NTf_2_]^−^ anion can provide more atoms (e.g., the oxygen in S=O and fluorine) to form hydrogen bonding with the hydroxyl group in glabridin, resulting in the higher efficiency. In addition, [C_4_mim][NTf_2_] possessed a lower viscosity (52 mPa·s), which facilitated mass transfer of the system and thus dissolution of glabridin in this IL.

Munakata et al. used 1-ethyl-3-methylimidazolium methylphosphonate ([C_2_mim][(MeO)HPO_2_]) as the extractant to separate citral from *Lemon Myrtle* [62]. The result showed that the yield of citral obtained using the mixture of IL and EtOAc (1:1, *w*/*w*) was 2.1 times higher than that obtained using ethanol due to the formation of hydrogen bonding between hydrogen in the IL anion and oxygen in citral. The IL could be recycled and reused for nine times for the extraction of citral. Dong et al. [63] combined computational techniques with experiment to explore the hydrogen bonding interactions between 1-butyl-3-methylimidazlium hexafluorophosphate ([C_4_mim][PF_6_]) and three bioactive homologues (i.e., genistein, daidzein and glycitein) as the model compounds. The binding energies between [C_4_mim][PF_6_] and the active components obtained by DFT calculations decreased in the following order: genistein > daidzein > glycitein, which was in accordance with the distribution coefficients *D* determined in the IL-water biphasic system. The results also showed that the hydrogen bondings are mainly intermolecular interactions in the IL-active component complexes, and [C_4_mim][PF_6_] can recognize the homologues with similar structures by forming different hydrogen bonding with the phenolic hydroxyl groups. In addition, it was demonstrated that the anion played a more important role in recognition of the homologues than the cation.

### 3.2. Functional Ionic Liquids

**Figure 7:**
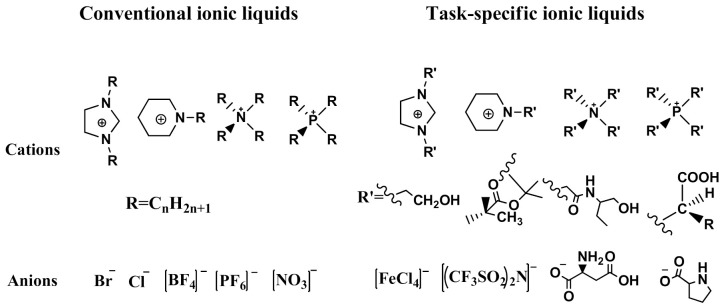
Structures of typical conventional ILs and TSILs.

One of the features of ILs is that their properties can be designed by changing the cation and/or anion structure to meet a specific requirement [64]. A series of functional ILs for natural product extraction have been designed, which could be divided into task-specific ILs (TSILs) and IL-derivatives, with the latter referring to porous ILs and molecularly imprinted ILs in this paper. With regard to natural product extraction, the extraction procedure performed with TSILs involved liquid-liquid extraction (i.e., LLE, ABS, or LPME), whereas when IL-derivatives are employed, micro-solid-phase extraction is used. Figure 7 demonstrates typical structures of conventional ILs and TSILs. In the following subsections, natural product extraction using these functional ILs will be introduced in detail.

#### 3.2.1. Magnetic Ionic Liquids

Although conventional ILs can be considered as alternatives to replace the flammable or toxic organic solvents employed in classical extraction, IL-based extraction typically requires centrifugation or phase separation to isolate the target compounds-enriched phase. One kind of ILs known as magnetic ILs (MILs) are proposed as extraction solvents to replace common ones in the presence of an external magnetic field [65,66,67,68]. MILs possess not only the properties of conventional ILs but also paramagnetic property through the introduction of a paramagnetic component which originates from the anion, the cation, or both [69,70]. According to their structures, magnetic ILs can be divided into organic MILs (e.g., 1-methyl-3-(4-oxo-4-(2,2,6,6-tetramethyl-1-oxyl-4-piperidoxyl)butyl)imidazolium chloride) [71] and MILs containing metal elements. The structures of some typical MILs of different anions and cations are listed in Table 1.

Compared with traditional magnetic fluids (magnetorheological fluids and ferrofluids), MILs possess excellent properties of traditional ILs, optical transparency and small-line width, which make them appropriate in many areas, including extraction, gas adsorption, magnetic separation, nanomaterial synthesis, catalysis and cellulose dissolution. The inherent magnetic responsivity of MILs was used to enhance the efficiency in the field of separation. Table 1 provides the extraction efficiency of triazine herbicides, phenols and acidic pharmaceuticals, aromatic sulfur compounds and sinomenine involving some typical MILs.

Wang et al. synthesized 1-hexyl-3-methylimidazolium tetrachloroferrate ([C_6_mim][FeCl_4_]) to separate triazine herbicides from vegetable oils. The results showed that the extraction capacities increased obviously when the amount of [C_6_mim][FeCl_4_] increased from 30 µL to 90 µL, and the maximum recoveries reached 114.2% when the amount of MIL was 90 µL. The magnetic ionic liquid-based dispersive liquid-liquid microextraction gave the limits of quantification of 4.33–4.91 ng/mL and limits of detection of 1.31–1.49 ng/mL for triazine herbicides. The recoveries were within the range of 81.8–114.2% and the relative standard deviations were less than 7.7% [72]. Chatzimitakos et al. [73] prepared MIL methyltrioctylammonium tetrachloroferrate ([N_8,8,8,1_][FeCl_4_]) and proposed a drop-breakup microextraction method to extract acidic pharmaceuticals and phenolic endocrine disrupters (bisphenol A, 17β-estradiol, 3-nitrophenol, etc.). The recoveries were within the range of 89–94%. Deng et al. [74] employed a hydrophobic MIL trihexyltetradecylphosphonium tetrachloroferrate(III) ([3C_6_PC_14_][FeCl_4_]) to extract phenolic compounds, and the concentration of pentachlorophenol could decrease from 7.8 μg/mL to 0.2 μg/mL. Hydrophobicity, along with hydrogen bonding of phosphorus atom in cation with hydroxyl group in phenols (P^…^H-O), played major roles in the selective extraction of phenols from aqueous solution under acidic conditions. Jiang et al. [75] synthesized a series of MILs, i.e., 1-*N*-butyric acid-3-methylimidazolium chloride/xFeCl_3_ ([C_3_H_6_COOHmim]Cl/xFeCl_3_; x = 0.5, 1, 1.5, 2), and measured their extraction efficiency for aromatic sulfur compounds (i.e., dibenzothiophene and its derivatives). The results showed that [C_3_H_6_COOHmim]Cl/2FeCl_3_ was highly active for the extraction of aromatic sulfur compounds and the extraction efficiency could reach 100% in 10 min under mild conditions. Li et al. [76] used MILs based on imidazolium cations and iron(III) anions for the ultrasonic-assisted extraction (UAE) of sinomenine from *Sinomenium acutum*. The extraction amount reached 10.57 mg/g under the optimized conditions. The recovery and the purity of sinomenine reached 81.3% and 82.6%, respectively, after purification. This hybrid method of MIL-UAE and reversed micellar extraction used for the extraction and purification of sinomenine was efficient, which had great potential for scale-up. Solubility of sinomenine in the MIL was higher compared with those in organic solvents because of the multi-interactions, such as hydrogen bonding, between MILs and sinomenine.

Magnetic extraction could eliminate the formation of emulsification, which frequently occurs in traditional extraction processes, making it inconvenient in recovery of the extraction solvent. The excellent performance of MILs in separation could be due to their ability to form hydrogen bonding, selective solvation capability and wide liquid range. Under the same conditions, the consumption of MILs for a certain separation process has been shown to be less, which is in line with the concept of “green chemistry”.

#### 3.2.2. IL-Derivatives

##### Porous Ionic Liquids

Porous polymers have been widely used in catalysis, gas separation, electrode materials, and so on [77,78]. Solidification of fluidic ILs into porous materials yields porous materials, which integrate the unique properties of ILs and porous polymers, such as low bulk density, enlarged surface areas and pore volumes, high chemical versatility, enhanced mechanical stability, and processability inherent to polymeric architectures [79,80,81].

**Figure 8:**
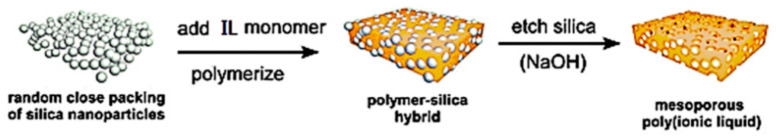
Schematic overview of the employed hard-templating method for synthesis of mesoporous poly(ionic liquid) [83]. Reprinted with permission of American Chemical Society.

The preparation methods of porous ILs mainly involve template synthesis (hard-templating and soft-templating) and template-free synthesis. The tri-block copolymer P123 (EO_20_PO_70_EO_20_) as the soft template and 1-allyl-3-vinylimidazolium IL as the monomer were used to synthesize ILs with tunable pore structure [82]. Wilke et al. synthesized a mesoporous PIL by a hard-templating method based on silica nanoparticles (Figure 8). First, silica nanoparticles with the average diameter of 25 nm were slowly dried to form mesoporous particles. Then, the mesoporous silica and 3-(4-vinylbenzyl)-1-vinylimidazolium bis(trifluoromethylsulfonyl)imide monomer polymerized in the presence of an initiator, and a transparent PIL-silica hybrid was fabricated. Finally, silica was removed by the addition of NaOH and mesoporous poly(ionic liquid) was obtained [83].

**Figure 9:**
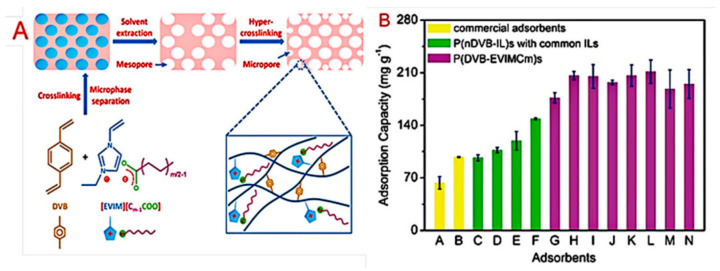
Synthetic route for mesoporous PILs and hypercrosslinked mesoporous PILs (**A**) and their adsorption capacity for mixed tocopherols (**B**) [84]. Reprinted with permission from Royal Society of Chemistry.

Suo et al. synthesized a family of anion-functionalized mesoporous PILs with 1-ethyl-3-vinylimidazolium laurate as the monomer and divinylbenzene as the crosslinker [84]. These porous copolymers (green bars in Figure 9) showed excellent adsorption capacities for tocopherols (211.45 mg/g) and high selectivity (i.e., selectivity of δ-tocopherol to α-tocopherol (S_δ/α_): 8.65; that of β- and γ-tocopherol to α-tocopherol (S_β&r/α_): 4.20) for bioactive tocopherol homologues, better than those of commercial adsorbent systems (yellow bars in Figure 9). The prepared mesoporous PILs, with carboxylate (-COO^−^) serving as stronger hydrogen bonding acceptors, possessed great affinity for tocopherols, which are scarce in these commercial resin adsorbents. Zhao et al. utilized poly(ionic liquid)-functionalized magnetic material to enrich eight pyrethroids in apples [85]. The extraction process only needed 1 min, which greatly decreased extraction time. The detection limit was from 0.24 to 1.99 ng/g, and the linearity range was 10–200 ng/g. Recoveries of targets in apple samples were in the range of 87.3–119.0%. The RSD for intraday precision ranged from 3% to 21.2% and that for interday precision was from 0.3% to 15.2%. The results showed that the proposed method was appropriate for extraction of pyrethroid residues in apple samples.

##### Molecularly Imprinted Ionic Liquids

Molecular imprinting is a promising technique widely used in the synthesis of molecularly imprinted polymers (MIPs) with specific molecular recognition properties [86,87]. Owing to their excellent properties, ILs have been utilized as the functional monomers for preparation of MIPs [88]. Generally speaking, IL-based MIPs involve multiple interactions between the analyte and ILs (i.e., π-stacking, electrostatic, hydrophobic interactions, and hydrogen bonding) [89,90,91]. IL-based molecularly imprinted polymers (IL-MIPs) have aroused widespread concern as recognition materials because of their easy preparation, predictable specific recognition capability, high stability, high binding capacities and water-compatibility [92]. They have been widely applied in solid-phase extraction, chemical sensor, affinity chromatography, enzyme-involved catalysis, and drug delivery [93,94].

**Figure 10:**
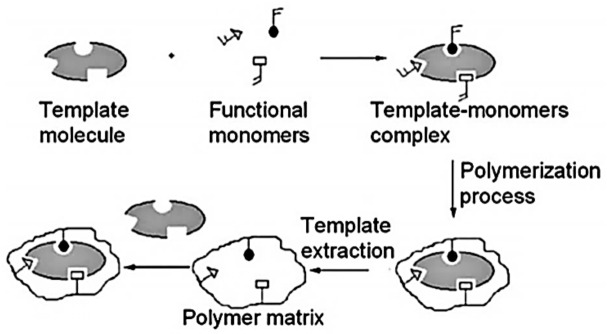
General scheme of preparation and recognition mechanism of MIPs [96]. Reproduced with permission of Elsevier.

IL-MIPs could be synthesized by copolymerization of ionic liquid functional monomer, template molecule and cross-linker in the presence of the initiator (Figure 10) [95,96]. When the template molecule is removed from the polymer, the IL-MIPs possess specific binding sites which are complementary to the template in shape, size and functional group orientation, so they can extract and separate the target substance from complex samples.

**Figure 11:**
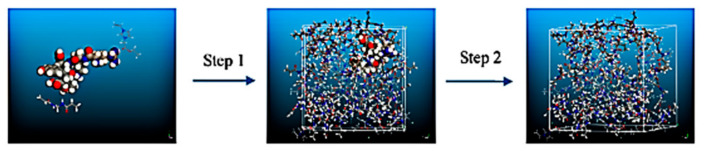
Schematic representation of the molecular imprinting of thymopentin. Specific binding cavities are generated using IL as the functional monomer [93]. Reprinted with permission of Elsevier.

Wang et al. prepared molecular imprinting membrane with IL 1-vinyl-3-ethyl acetate imidazolium chloride as the functional monomer [93]. After polymerization (step 1 in Figure 11) and removal of the template (step 2), binding sites containing template-specific shapes were left in the imprinting polymer. The obtained membranes were used to separate thymopentin from crude samples. The experimental results showed that solid-phase extraction equipment filled with molecularly imprinted membranes had higher separation and identification performance for thymopentin. The simulation results indicated that the IL monomer showed higher interaction energy with thymopentin than that with other components in the samples.

Yuan et al. utilized 1-allyl-3-methylimidazolium bromide and acrylamide as co-functional monomers and (*Z*)-6-fluoro-3-(hydroxymethylene)-thiochroman-4-one as the template molecule to prepare IL-based MIPs in order to extract (*Z*)-3-(chloromethylene)-6-flourothiochroman-4-one [97]. Compared with conventional sorbents and IL-non-imprinted polymers, IL-MIPs possessed excellent purification and adsorption abilities for (*Z*)-3-(chloromethylene)-6-flourothiochroman-4-one, which could be attributed to interactions between the IL monomer with the fluoride and chlorine aoms in (*Z*)-3-(chloromethylene)-6-flourothiochroman-4-one by ion exchange. Tang et al. synthesized MIPs based on a ternary deep eutectic solvent (DES) to recognize polyphenols [98]. The DES is a kind of IL or pseudo IL and was obtained by combining a quaternary ammonium salt as the hydrogen bonding acceptor and the template molecule polyphenols as the hydrogen bonding donor. The ternary DES-based MIPs possessed better adsorption and specific recognition capacity compared with C_8_, C_18_ or non-imprinted polymer adsorbents. The isotherms and kinetics of recognition process by the ternary DES-based MIPs were utilized to study its interactions with polyphenols. It was confirmed that the key interactions were mainly chemical interactions (i.e., new bonding formation, electron exchange, etc.) between the functional groups on the MIPs surface and OH, COOH, related ions in polyphenols. Chen et al. utilized 3,4-dihydroxybenzenepropanoic acid as the template molecule and 1-ally-3-vinylimidazolium chloride as the functional monomer to prepare poly(ionic liquid)-based MIP for the extraction of phenolic acids from fruit juice and beer samples [99]. Compared with reported approaches, this method showed high selectivity and satisfactory extraction sensitivity for the separation of template molecule from its homologues. 

## 4. Conclusions and Outlook

ILs as extraction solvents have received extensive attention in the separation of natural products because of their excellent performance, thereby solving some problems facing by traditional solvents. In this review, recent progress on the understanding of the special interaction, i.e., hydrogen bonding, in ILs was reviewed. Extraction of various natural products by ILs and the roles of multi-interactions in this process were summarized. The hydrogen bonding is shown to be one of the major driving forces resulting in excellent extraction efficiency of ILs in natural product extraction. More IL-based functional materials that can enhance the extraction of natural products are in demand. In order to meet the specific extraction requirements of natural products, it is necessary to adjust the structures and properties of IL cations and anions. The key point in design of IL-based materials for the extraction of natural product is the interactions between the functional groups and target substances. However, current research on extraction and separation with IL-based materials is still in the initial stage, and lots of issues have not been deeply understood to meet industrial demands. For instance, the data on physicochemical properties of ILs are still not sufficient and their extraction mechanisms need more exploration. In addition, there exist production cost, residues and safety problems of ionic liquids. In order to put IL-based separation technology into commercial use, relevant studies are in urgent need.

Firstly, the combination of different cations/anions will generate lots of ILs, including functionalized ones. To develop efficient and environmentally friendly separation process with IL-based materials, one of the challenges is to systematically and comprehensively understand the relationship among IL structures-physicochemical properties-extraction performance, which could guide the design of suitable ILs or IL-based materials. The combination of molecular simulation with experimental characterizations is an efficient method to reveal the structure-property relationship and biomaterials extraction mechanism, which deserves people’s attention, especially young scientists.

Secondly, the cost of ILs is still higher than that of traditional solvents, which restricted their large scale use in industry. Most ILs are synthesized in laboratory scales with complex synthesis and purification steps. The development of synthetic method with less preparation steps and cheaper raw materials, and increase of the production scale can reduce the cost of ILs to some extent. Moreover, an appropriate balance between the cost and the performance of ILs should be considered.

Additionally, with the enhancement of environmental awareness, issues related to the use of ILs, such as residue, long-term stability, safety and toxicity, should also be a hot topic in the future. The development of biocompatible and biodegradable ILs for use in the pharmaceutical field is also a promising direction. It is believed that ILs will be more widely employed in many fields, including natural product extraction, and more people are welcomed to join in related work.

## Figures and Tables

**Table 1:** Structures and extraction efficiencies of typical magnetic ILs.

MIL		Components Extracted	Recoveries (%)	Ref.
Cation	Anion			
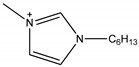	[FeCl_4_]^−^	Triazine herbicides	81.8–114.2	[72]
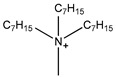	[FeCl_4_]^−^	Phenols and acidic pharmaceuticals	89–94	[73]
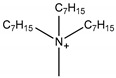	[FeCl_4_]^−^	Phenolic compounds	-	[74]
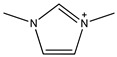	[Cl/xFeCl_3_]^−^	Aromatic sulfur compounds	100	[75]
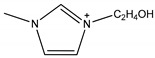	[FeCl_4_]^−^	Sinomenine	81.3	[76]

## Data Availability

Not applicable.

## Abbreviations

[C_4_mim][BF_4_]	1-Butyl-3-methylimidazolium tetrafluoroborate
[Bim][NTf_2_]	1-Butylimidazolium bis(trifluoromethylsulfonyl)imide
[C_4_mim][NTf_2_]	1-Butyl-3-methylimidazolium bis(trifluoromethylsulfonyl)imide
[Bmpyr]Br	Butylmethylpyrrolidinium bromide
[C_4_mim][PF_6_]	1-Butyl-3-methylimidazlium hexafluorophosphate
[NEt_2_(HE)_2_]Br	Bihydroxyethylbiethylammonium bromide
[(CH_3_)_2_NH_2_]Br	Dimethylammonium bromide
[EPy]Br	Ethylpyridinium bromide
[C_2_mim][(MeO)HPO_2_]	1-Ethyl-3-methylimidazolium methylphosphonate
[EtOHmim][NTf_2_]	1-(2-Hydroxyethyl)-3-methylimidazolium bis(trifluoromethylsulfonyl)imide
[C_4_C_11_OHim][CF_3_SO_3_]	1-Butyl-3-(11-hydroxyundecyl)imidazolium trifluoromethanesulfonate
[C_6_mim][FeCl_4_]	1-Hexyl-3-methylimidazolium tetrachloroferrate
[EtOHmim][BF_4_]	1-(2-Hydroxyethyl)-3-methylimidazolium tetrafluoroborate
[L-Pro]_2_[SO_4_]	L-Proline sulfate
[Memo]Br	Methylethylmorpholinium bromide
[N_8,8,8,1_][FeCl_4_]	Methyltrioctylammonium tetrachloroferrate
[Mepip]Br	Methylethylpiperidinium bromide
[N_3111_][NTf_2_]	N-trimethyl-N-propylammonium bis(trifluoromethylsulfonyl)imide
[HOOC(CH_2_)_3_mim][NTf_2_]	1-N-butyrate-3-methylimidazolium bis(trifluoromethylsulfonyl)imide
[C_3_H_6_COOHmim]Cl/xFeCl_3_	1-N-butyric acid-3-methylimidazolium chloride/xFeCl_3_
[(CH_3_)_3_NH][NO_3_]	Trimethylammonium nitrate
[(CH_3_)_4_N][NO_3_]	Tetramethylammonium nitrate
[3C_6_PC_14_][FeCl_4_]	Trihexyltetradecylphosphonium tetrachloroferrate(III)
[(CH_3_)_3_NH]Br	Trimethylammonium bromide
